# Inference of hierarchical regulatory network of estrogen-dependent breast cancer through ChIP-based data

**DOI:** 10.1186/1752-0509-4-170

**Published:** 2010-12-17

**Authors:** Fei Gu, Hang-Kai Hsu, Pei-Yin Hsu, Jiejun Wu, Yilin Ma, Jeffrey Parvin, Tim H-M Huang, Victor X Jin

**Affiliations:** 1 Department of Biomedical Informatics, The Ohio State University, Columbus, USA; 2 Human Cancer Genetics Program, The Ohio State University, Columbus, USA

## Abstract

**Background:**

Global profiling of in vivo protein-DNA interactions using ChIP-based technologies has evolved rapidly in recent years. Although many genome-wide studies have identified thousands of ERα binding sites and have revealed the associated transcription factor (TF) partners, such as AP1, FOXA1 and CEBP, little is known about ERα associated hierarchical transcriptional regulatory networks.

**Results:**

In this study, we applied computational approaches to analyze three public available ChIP-based datasets: ChIP-seq, ChIP-PET and ChIP-chip, and to investigate the hierarchical regulatory network for ERα and ERα partner TFs regulation in estrogen-dependent breast cancer MCF7 cells. 16 common TFs and two common new TF partners (RORA and PITX2) were found among ChIP-seq, ChIP-chip and ChIP-PET datasets. The regulatory networks were constructed by scanning the ChIP-peak region with TF specific position weight matrix (PWM). A permutation test was performed to test the reliability of each connection of the network. We then used DREM software to perform gene ontology function analysis on the common genes. We found that FOS, PITX2, RORA and FOXA1 were involved in the up-regulated genes.

We also conducted the ERα and Pol-II ChIP-seq experiments in tamoxifen resistance MCF7 cells (denoted as MCF7-T in this study) and compared the difference between MCF7 and MCF7-T cells. The result showed very little overlap between these two cells in terms of targeted genes (21.2% of common genes) and targeted TFs (25% of common TFs). The significant dissimilarity may indicate totally different transcriptional regulatory mechanisms between these two cancer cells.

**Conclusions:**

Our study uncovers new estrogen-mediated regulatory networks by mining three ChIP-based data in MCF7 cells and ChIP-seq data in MCF7-T cells. We compared the different ChIP-based technologies as well as different breast cancer cells. Our computational analytical approach may guide biologists to further study the underlying mechanisms in breast cancer cells or other human diseases.

## Background

Global level profiling of in vivo protein-DNA interactions using ChIP-based technologies has evolved rapidly in recent years, from hybridization with spotted or tiling microarray (ChIP-chip) [1-4], to SAGE-like tags (ChIP-SAGE) [5] or pair-end tag sequencing (ChIP-PET) [6], to current massively parallel sequencing (ChIP-seq) [7-10].

Estrogen-mediated gene regulation is such a challenging question that it may require powerful genome-wide profiling tools like ChIP-based technologies. In breast cancer cells, ERα can mediate genomic transcription regulation with nuclear initiated steroid signalling and non-genomic activation of various protein kinase cascades [11]. In the classical genomic pathway, estrogen receptor binds to estrogen response elements (ERE) at the regulatory region of the target genes and recruits co-activators or co-repressors to modulate gene transcription [12]. The non-classical genomic pathway does not require ERE but mediates transcription by the interactions of ERα with other proteins such as AP1 [13], NF-kB [14], SP1 [15,16] and others. At molecular level, we need to identify those genes targeted and regulated by estrogen receptors, and the more challenging task is to delineate the architectures and the underlying mechanisms of such regulation. Estrogen receptors, once activated, may induce increased or decreased transcription of its numerous targets, which have been investigated by expression arrays [17,18]. In some recent publications, profiling the distribution of ERα by ChIP-seq ChIP-PET and ChIP-chip indicated a highly complicated regulation network involved with both ERα and other relative transcription factors [17-19]. For instance, only a small portion of ERα binding sites were located in the promoter regions of known genes and many unforeseen binding sites could be far away from the TSS, up to 50-100 kb. It was also found that a large number of transcription factors had binding sites co-enriched with ERα binding sites, which indicated a close collaboration between ERα and other factors. All these findings support the evolving concept of estrogen receptor regulation from the conventional interaction between ERE and ERα to the long-range chromatin loop [20].

Tamoxifen is one of selective ER modulators (SERMs) and is widely used to block ERα function for breast cancer treatment [11]; however, this endocrine therapy is limited by the onset of drug resistance. Tamoxifen resistance could be induced through both genomic and non-genomic estrogen pathways mentioned above and understanding estrogen regulation network will offer therapeutic advantages. Our recent expression array study showed that, in breast cancer cells with acquired tamoxifen resistance, different groups of genes were targeted by estrogen treatment compared with the parental cells [20]. Delineating the changed architectures of the ERα regulation network in tamoxifen resistance cells by ChIP-based assays may provide direct and useful information on tamoxifen resistance.

In this study, we collected three public available ChIP-based datasets--ChIP-chip, ChIP-PET and ChIP-seq for ERα binding sites in breast cancer MCF7 cells upon estrogen exposure, which also include RNA polymerase II (Pol-II) binding sites in these cells since the binding of Pol-II could provide direct information of potential transcription activation. We then applied computational approaches to investigate the hierarchical regulatory information for ERα regulation in MCF7 cells. We have been able to construct hierarchical regulatory networks with target hubs and have established the regulatory pathways between TFs and genes. We also have applied ChIP-seq technology to systematically compare the estrogen-mediated regulatory information between MCF7 and MCF7-T cells.

## Results

### Whole-Genome-Wide localization of *in vivo *ERα/Pol-II binding peaks to genes in breast cancer cells

It is well recognized that the transcription is driven by Pol-II and the general transcriptional machinery; therefore, it is likely that the genes (or promoters) bound by ERα may not be transcribed without Pol-II binding. As such, we need to use the whole-genome-wide binding peaks information combining ERα with Pol-II antibodies.

The peak-calling procedure was described in the **Methods **section. The number of ChIP peaks from ChIP-seq is 12,516 for ERα and 13,261 for Pol-II upon E2-treated compared to the vehicle control, the number from ChIP-PET is 14,703 for ERα and 13,133 for Pol-II, the number from ChIP-chip is 10,409 for ERα and 11,455 for Pol-II (Additional file 1, Table S1).

Our previous studies [20-23] and others [24-28] have tried to establish and characterize the molecular mechanisms of estrogen-dependent breast cancer cells. In order to further elucidate the more detailed underlying mechanisms, we then created a whole-genome-wide localization of *in vivo *ERα binding peaks in the MCF7. We located these identified ERα and Pol-II binding peaks relative to a known annotated gene from the RefSeq database (UCSC HG18 Assembly). Our results showed that 18% for ChIP-seq, 44% for ChIP-PET, 45% for ChIP-chip of Pol-II binding peaks are located within Promoter regions (defined as 2 kb upstream to 2 kb downstream, including 5' Core, 5'TSS and WithinGene Core regions) of a known transcription start site (5'TSS) (Additional file 1, Table S1 and Additional file 2, Figure S1). We also found that a relatively small number of ERα binding peaks are located in a known 5'TSS region (9% for ChIP-seq, 6% for ChIP-PET, 6% for ChIP-chip). A big portion of ERα binding peaks are located within intra-genic regions (38% for ChIP-seq, 37% for ChIP-PET, 58% for ChIP-chip) as well as gene desert regions (14% for ChIP-seq, 20% for ChIP-PET, 12% for ChIP-chip), 100 kb far away from known 5'TSSs and 3'TSSs (Additional file 2, Figure S1B). While our location analysis confirms the results from previous studies [17-19] where the data were collected, our comparative analysis further showed ERα binding patterns are essentially similar in which the majority of ERα binding peaks are outside of proximal promoter regions regardless of different ChIP technologies. Our analysis also suggests that most ERα associated genes may be regulated by the long-distance interaction between ER-bound distal enhancer and proximal promoter regions. We also found that some portions (18% for ChIP-seq, 17% for ChIP-PET, 11% for ChIP-chip) of Pol-II binding peaks are located in the 3' ends (including 3' Core, 3' Proximal and 3' Distal regions). This finding might imply some alternative transcripts are transcribed in the 3' end and is consistent to the previous report of alternative promoters identified in the 3' end [29]. This could also be simply due to either stalled RNA Pol II that has finished transcription or loops formed with the promoters as has been proposed [30].

### Regulation of ERα target gene expression in MCF7 cells

**Figure 1:**
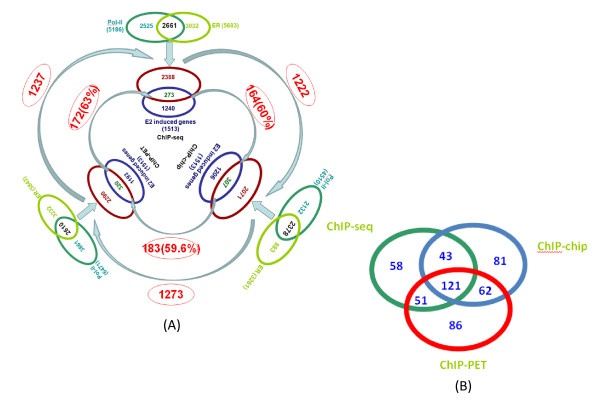
**Summary of correlations of identified ERα, Pol-II binding peaks with gene expression profile after E2-induced in MCF7 cells**. (A) Genes with both ER and Pol-II peak binding in the gene region (between 100 kb upstream of TSS and 100 kb downstream of 3' UTR), 2661 (273 common genes overlapped with gene expression data) for ChIP-seq, 2610 (320 common genes overlapped with gene expression data)for ChIP-PET dataset and 2378 (307 common genes overlapped with gene expression data) for ChIP-chip dataset, respectively. 172(~63%) common genes were found between ChIP-seq and ChIP-PET, this number was higher than the common genes number between ChIP-seq and ChIP-chip (164, ~60%) and common genes number between ChIP-chip and ChIP-PET (183, ~59.6%). (B) The overlapped number of genes of three different technologies.

We then systematically compared three different ChIP based technologies. We aim to correlate 1,513 E2-induced genes identified by Carroll et al. [17] to ERα and Pol-II binding peaks identified from different ChIP-based datasets. For Pol-II binding peaks, we only considered those peaks within proximal upstream regions (less than 10 kb) relative of TSS, within intra-genic regions, and within proximal downstream regions (less than 10 kb) relative of TTS. For ERα binding peaks, we considered all binding peaks except those in the gene desert regions (larger than 100 kb away from a TSS). For ChIP-seq data, it turned out to have a total of 12,516 ERα binding peaks corresponded to 5,693 annotated genes and 13,261 Pol-II binding peaks corresponded to 5,186 genes. And 2,661 genes were identified to have both Pol-II and ERα binding peaks in ChIP-seq dataset (Figure 1A, top panel). Among these 2,661 genes with enriched double (ERα and Pol-II) binding peaks, only 273 of them overlapped with 1,513 E2-induced genes in MCF7 show differential expression (Figure 1A, top panel). Surprisingly, a majority (1,240 of 1,513) of genes in ChIP-seq dataset with differential expression lack ERα and Pol-II binding sites. After examining the correlation of ERα and Pol-II identified by ChIP-chip dataset in MCF7, we obtained very similar results, where 307 genes have both Pol-II and ER enriched binding peaks. (Figure 1A, bottom-right panel). In order to exclude different experiments' discrepancy, we also compared the ERα and Pol-II binding data identified by ChIP-PET, and E2-induced expression data in MCF7. Similarly, only 320 common genes were obtained (Figure 1A, bottom-left panel). The overlap of gene number of three different technologies was shown in Figure 1B. Overall, the similar findings in all three ChIP-based dataset seem to indicate that ERα binding and E2-mediated Pol-II binding in most of ERα direct targets may not necessarily lead to detectable activated or repressed expression changes. Instead, they may be in paused states as the previous reports suggested [18,31,32]. Meanwhile, it also seems that a majority of E2-induced expression changes could be caused by downstream estrogen effect and may have not direct and close correlation with ERα-recruited Pol-II complex for transcription initiation [33].

We also found that 164 genes (~60%) are common between ChIP-seq and ChIP-chip (Figure 1A) while 183 (~59.6%) common genes between ChIP-chip and ChIP-PET (Figure 1A). A comparison of ChIP-seq and ChIP-PET has shown that they have 172 (~63%) common genes (Figure 1A).

### *De Novo *identification for ERα binding sites and its binding partners in MCF7 cells

One possible scenario to explain why the majority of ERα direct targets are not activated or repressed is that they may lack certain binding TF partners serving as helpers for ERα to co-regulate these genes. Thus we applied the *de novo *motif discovery approach (ChIPMotifs) [34,35] developed in our previous study to identify the known or novel ERα TF partners. ChIPMotifs is online software designed for searching the most significant motifs in given peaks based on the known factor motifs in TRANSFAC [36] and JASPAR [37] databases.

**Figure 2:**
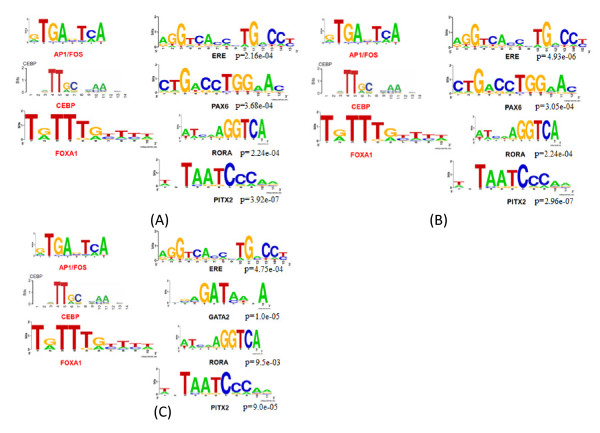
**List of transcription factor motifs identified by our *de novo *ChIPMotifs approach**. (A) ChIP-seq, (B) ChIP-PET, and (C) ChIP-chip.

In this study, for each ChIP-based dataset, we selected the top 2000 peaks with high scores (enrichments) as the input data for ChIPMotifs. After running ChIPMotifs, ERE, PAX6, PITX2 and RORA were identified in ChIP-seq data; ERE, PITX2, RORA and GATA2 were identified in ChIP-chip data; RORA, PAX6, PITX2 and ERE were identified in ChIP-PET data (Figure 2). We failed to identify several TFs (CEBP [38], FOS (AP1) [39], FOXA1 [17]) reported to be associated with ERα in previous studies. One possible reason is that our *de novo *ChIPMotifs first *ab initio *identify motifs at a set of relatively short sequences (~300 - 500 bp), then find possible matched TFs from the TRANSFAC database after obtaining significant motifs, therefore, this might miss some co-TFs if they locate outside 500 bp distance from ERα. But if we used longer sequences, that might lose the specificity for the identified motifs such as missing identifying ERE. Regardless, we chose to include these three co-TFs (CEBP, FOS and FOXA1) in our further analysis. These TFs' Seq-LOGOs in ChIP-seq data, ChIP-chip data and ChIP-PET data were shown in Figure 2. Further analysis of these data indicates that in addition to ERE, there are two TF motifs common among ChIP-seq data, ChIP-chip data and ChIP-PET data--PITX2 and RORA (Figure 2).

### ERα regulatory network in estrogen-dependent MCF7 cells

Previous studies have shown ERα regulates its target genes through three major binding models a) direct binding to ERE (estrogen response element); b) indirect binding, through which it binds to other TF partners which bind to DNA; and c) co-occurrent binding, where both ERα and other TF partners bind to their own specific DNA motifs (Additional file 3, Figure S2) [20-22,40,41].

**Figure 3:**
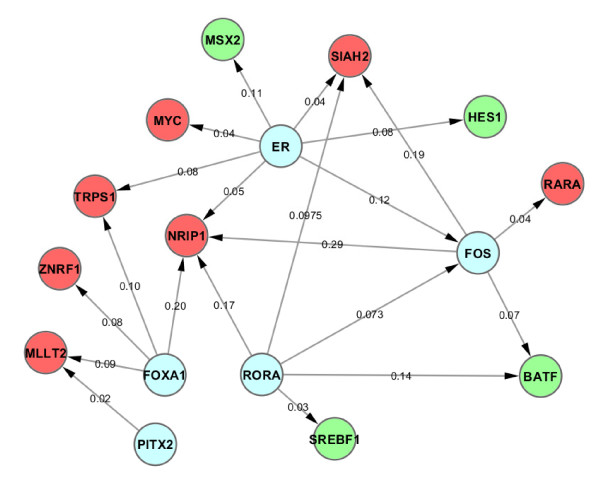
**The regulatory network for E2 treated MCF7 cells, combining with 3 different ChIP-based datasets**. Red nodes represented for up-regulated genes, green nodes represented for down-regulated genes, and blue nodes represented for Hub TFs.

In order to understand how the regulatory network responds to E2 treatment in three ChIP-based technologies, we here integrated the ChIP data with the time series of E2 induced gene expression data. In order to determine if a gene is differentially expressed, we used the difference of expression levels between time point 12 hr and 0 hr, positive value as up-regulated and negative value as down-regulated. Thus all genes in the networks were differentially expressed and with ERα and PolII binding ChIP-peaks (as mentioned in "**Regulation of ERα target gene expression in MCF7 cells**" section, the ERα peak location is between 100 kb upstream of 5'TSS and 100 kb downstream of 3'TSS, while the Pol-II peak location is between 10 kb upstream of 5'TSS and 10 kb downstream of 3'TSS. The ERα peak and Pol-II peak do not necessarily be overlapped, but they must be located in the same gene). Furthermore, for the transcriptional regulatory network (as a part of gene regulatory network), only transcription factors were used for the network construction (called normal TFs). ERα binding peaks associated with those normal TFs were further scanned by Hub TF (the TF which motif was enriched in top 2000 ERα binding peaks of the genes with both ERα and Pol-II binding sites, and was identified from the previous section "*De Novo *identification for ERα binding sites and its binding partners") PWMs to determine if there is any connection between a Hub TF and a normal TF. A shuffling test was performed to test the reliability of each connection of the network. The resulted regulatory networks were thus constructed and topologically visualized using Cytoscape [42] software platform (see **Methods **section and Figure 3). In the network, all the normal/Hub TFs were represented as a node (red nodes represented for up-regulated genes, green nodes represented for down-regulated genes, and blue nodes represented for Hub TFs), and all the connections were represented as edge between 2 nodes (Figure 3). An edge has a direction, where it starts from Hub TFs to normal TFs. The edge between a Hub TF PWM (ex: FOXA1) and a normal TF differentially expressed following estrogen stimulation (ex: MYC) means that a motif of Hub TF (i.e.: FOXA1) is found within ERα peak region(s) associated with another TF gene (i.e: MYC). Since every normal TF was with both ERα and Pol-II peaks, the edge represented for the possible direct/indirect binding of Hub TF (ex: FOXA1) together with ERα to regulate normal TF (i.e: MYC).

In summary, 40 nodes (Additional file 4, Figure S3A) were identified for ChIP-seq data, 50 for ChIP-PET and 37 for ChIP-chip (Additional file 4, Figure S3B, C). A comparison of the three regulatory networks shows that there are 6 common hubs (ER, FOS, RORA, FOXA1, CEBP and PITX2) and 16 (~40%) common targeted TFs (Additional file 5, Table S2) in MCF7. The final combined ERα regulatory network for MCF7 cell from all three ChIP-based datasets was shown in Figure 3.

### ERα regulatory pathway analysis in estrogen-dependent MCF7 cells

**Figure 4:**
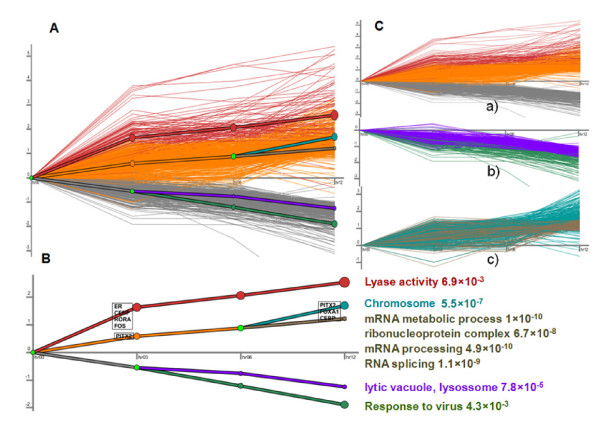
**Regulatory pathway analysis on the dataset after combining with all three ChIP-based datasets**. A) The time series gene expression data of E2 induced genes superimposed with the regulatory pathway map produced by DREM using the gene expression profile as well as ERα binding sites and Pol-II binding sites. The bright green nodes indicate split points where the sets of expression of genes diverge. B) Paths out of splits are annotated with TFs determined by DREM to be associated with the genes assigned to the path at a score <0.1. The GO annotations for the genes in 5 of the paths are shown at the right with their p-values. C) The genes traversing the 3 splits are shown with (a) corresponding to the split at 0-hr, (b) corresponding to the split at 3-hr and (c) corresponding to the split at 6-hr.

We next applied DREM program to model, analyze, and visualize dynamic gene regulatory functions [43]. The maps would potentially infer major bifurcation events in the time series expression data and transcription factors responsible for them. Take the ChIP-seq data as example, we used 6 ERα associated TF partners--RORA, PITX2 and PAX6 were identified by *de novo *ChIPMotifs from our data, and CEBP, FOS (AP1) and FOXA1 were known TF partners of ERα. After integrating the TF-DNA interactions data with 1,513 genes (see **Methods **section), a total of 5 paths were clearly diverging for these genes (Additional file 6, Figure S4 (I)). The same TF partners were used in ChIP-PET dataset (Additional file 6, Figure S4 (II)). The TF PAX6 was replaced with GATA2 in ChIP-chip dataset (Additional file 6, Figure S4 (III)). All the TF partners were selected based on the ChIPMotifs results (see **Results **section "*De Novo *identification for ERα binding sites and its binding partners"). A final combined regulatory pathway map obtained from three ChIP data showed that genes were traversing the 3 splits are shown with (a) corresponding to the split at 0 hr, (b) corresponding to the split at 3 hr and (c) corresponding to the split at 6 hr (Figure 4). The paths out of the splits were annotated with TFs determined by DREM to be associated with the genes assigned to the path at a score < 0.1. The GO annotations for genes in 5 of the paths were shown at the right with their p-values (Figure 4). Our finding showed E2 mediated a set of repressed genes with direct ERα binding sites where they are overrepresented with lytic vacuole/lysossome (p = 7.8 × 10^-5^). We further revealed a new estrogen-mediated up-regulated path requiring a TF partner PITX2, and the function of this set of genes is associated with ribonucleoprotein complex (p = 6.7 × 10^-8^), mRNA metabolic process (p = 1 × 10^-10^), mRNA processing (p = 4.9 × 10^-10^) and chromosome (p = 5.5 × 10^-7^). Our analysis also showed other paths with less significant genes: Lyase activity with a p-value of 6.9 × 10^-3 ^(RORA, FOS and CEBP), response to virus with a p-value of 4.3 × 10^-3^. We also found that the TF FOS, CEBP, FOXA1 and PITX2 are related to the up-regulated genes, while FOXA1 was shown up in both up and down-regulated genes. The gene lists of five paths were shown in Additional file 7, Table S3. The relationship of the 6 TFs (ERα, RORA, FOS, FOXA1 PITX2 and CEBP) and their related genes were shown in Additional file 8, Table S4.

### A comparison of MCF7-T and MCF7 cells

To further understand the difference of the ERα associated gene regulatory information between MCF7 and MCF7-T, a tamoxifen-resistant breast cancer cell line, we conducted ERα and Pol-II ChIP-seq experiments in MCF7-T cells (see **Methods **section). Around 22 million reads were obtained for Pol-II control data, and 19 million reads in Pol-II E2-treated data. For ERα antibody, the number is 14 million for control data and 16 million for E2-treated data (Table 1). After uniquely mapping to the human genome, the reads number reduced to 7 million for Pol-II control data, 5 million for Pol-II E2 treated data, 5 million for ERα control data and 4 million for E2 treated data, respectively (Table 1). In general, 25-30% reads were uniquely mapped to the human genome.

**Table 1:** A summary of binding peaks of Pol-II and ERα in control and E2-treated MCF7-T cells identified by ChIP-seq.

TFs	Cell conditions	Reads	Unique Mapped Reads
Pol-II	Control	22,168,614	7,434,175
	
	E2-treated MCF7-T	19,573,995	5,715,418

ERα	Control	14,075,733	5,161,146
	
	E2-treated MCF7-T	16,401,648	4,445,403

**Figure 5:**
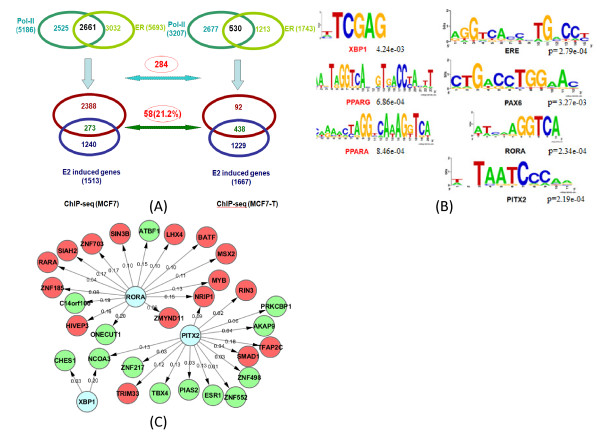
**Peak number, Motif and Regulatory network of MCF7-T cells**. (A) Comparison of common genes between MCF7 and MCF7-T cells in ChIP-seq dataset. Genes with both ER and Pol-II peak binding in the gene region (between 100 kb upstream of TSS and 100 kb downstream of 3' UTR), 2661 (273 common genes overlapped with gene expression data) for MCF7 cells, 530 (438 common genes overlapped with gene expression data) for MCF7-T cells, respectively. 58(~21.2%) common genes were found between MCF7 and MCF7-T cells. (B) Transcription factor motifs identified by our *de novo *ChIPMotifs approach for MCF7-T cells. (C) The Regulatory network for E2 treated MCF7-T cells.

In MCF7-T cells, 3,596 ERα binding peaks corresponded to 1,743 annotated genes and 4,644 Pol-II binding peaks corresponded to 3,207 genes were found. 530 genes were identified to have both Pol-II and ERα binding peaks (Figure 5A). 438 of them show differential expression overlapped with 1,667 E2-induced genes identified in MCF7-T expression profile analysis (Figure 5A). Similar to the MCF7 cells, a majority (1,229 of 1,667) of genes with differential expression lack ERα and Pol-II binding peaks.

Since we used ChIP-seq technology for MCF7-T cells, we compared the number of ERα regulated genes between MCF7-T and MCF7 cells using ChIP-seq technology. To illustrate the difference of these two cell lines, we first compared the overlapped number of genes with ERα binding peak and Pol-II binding peak, respectively. We found that 1075 genes were overlapped with ERα binding peak for both MCF7 and MCF7-T cell lines, and this number is 1508 for Pol-II binding peak. We also found that only 58 (~21.2%) common genes (Figure 5A) between these two breast cancer cells. This low overlapped number demonstrated that ERα targeted different sets of genes in these two cancer cells.

We next applied the same computational analytical approach to examine the ERα regulated network in MCF7-T cells. We started with training the top 2000 ERα binding peaks with high scores (enrichments) by the ChIPMotifs, a total of 7 TFs were identified, including ERE, PAX6, PITX2, RORA, XBP1, PPARG and PPARA (Figure 5B). Of them, ERE, PAX6, PITX2 and RORA are the same as the ones we identified in ChIP-seq data of MCF7 cells.

The ERα associated transcriptional regulatory network in MCF7-T cells was then constructed and visualized as shown in Figure 5C, including 32 nodes. A comparison of the networks between MCF7-T and MCF7 cells (using ChIP-seq dataset) showed that there are 2 common Hub TFs (RORA and PITX2) and 8 (about 25%) common targeted TFs. However, in MCF7 cells we found that there are 6 common Hub TFs and 16 (about 40%) common targeted TFs among different ChIP technologies. Taken together, our results strongly suggested that E2 induces a different ERα associated regulatory mechanism in MCF7-T cells compared to MCF7 cells, in other words, a rewired ERα regulation network in tamoxifen resistance cells.

## Discussion

In this study, we applied computational approaches to analyze and integrate three ChIP-based datasets and one time-series gene expression data to investigate the dynamic regulatory information for ERα in estrogen-dependent breast cancer MCF7 cells. Our studies not only compared the results of three ChIP-based protocols, but also have inferred the regulatory network and pathway for E2 induced MCF7 cells. Moreover, we used the same approach to compare the difference between MCF7 and MCF7-T cells.

Our comprehensive analysis of the estrogen-mediated regulatory network in MCF7 indicated that the three ChIP-based technologies have similar peak distribution patterns for the same antibody. However, for different antibody (ERα and Pol-II), the binding preference is totally different. Pol-II tends to binding to the promoter region, while ERα has no specific preference.

Our analysis (Figure 1A) also showed that there were more common regulated genes (in percentage) between ChIP-seq data and ChIP-PET data than ChIP-chip data. This may be due to a similar sequencing-based technology used for ChIP-seq and ChIP-PET, but an array-based technology used for ChIP-chip data. The regulatory network analysis indicates some common regulatory Hub TFs were formed in response to estrogen signaling and may lead to the same regulatory paths in MCF7. We also found that there are ~40% common TFs regulated by ERα among three different ChIP-based technologies.

We also compared the regulatory network in MCF7 constructed by our approach with another method--ReMoDiscovery [44]. We used 0.9 (for Motif threshold), and 0.5 (for expression correlation threshold) as the input threshold. Total 26 (of 42) nodes were overlapped between our method (Additional file 4, Figure S3A) and ReMoDiscovery (Additional file 9, Table S5).

In Figure 4, all the genes were with both ERα and Pol-II peak binding, and differentially expression between 12 hours of E2 treat and control. In Figure 4A, the genes were clustered based on the expression values. Each thin line meant the gene expression data (relative value compared with the first time point) at different time point. The genes were classified into 3 groups (the second time point), 4 groups (the third time point) and 5 groups (the fourth time point), respectively. The thick lines corresponded to the gene expression trend of each group. In Figure 4B, the important Hub TFs (Hub TFs with statistically significant number of motifs in the ERα binding peaks of groups of genes) were identified for each group at every time point (4 Hub TFs for time point 2, group 1; 1 for time point 2, group 2 and 3; 3 for time point 4, group 2). The 5 groups of genes were also showed significantly different gene functions. We listed the functions and *p*-values. Figure 4C (a) showed both up and down regulated genes, (b) corresponded to down regulated genes, and (c) corresponded to up regulated genes. A TF-gene relationship table is prepared as the input of DREM (Additional file 8, Table S4). In this table, 1 represented motif of Hub TF existed in the peak region of a gene.

**Figure 6:**
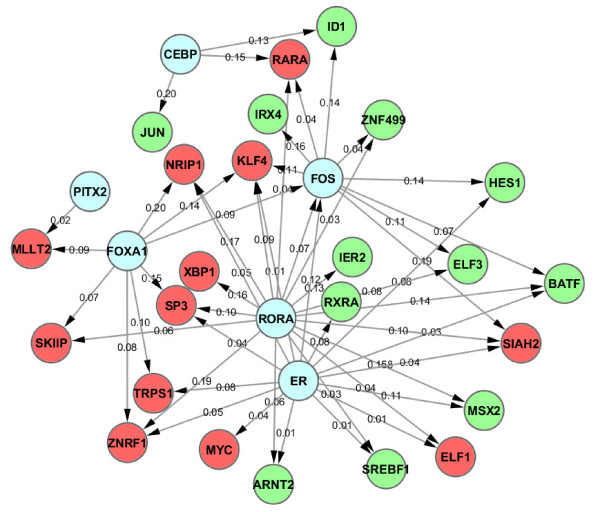
**The Regulatory network for E2 treated MCF7 cells, combining with 2 different ChIP-based datasets: ChIP-seq and ChIP-PET**. Total 30 nodes (TFs) were found in the network. This number is approximately 60% of the nodes in the network of ChIP-seq/ChIP-PET only.

Since a totally different technology is used for ChIP-chip data, in order to reduce the inconsistency caused by the technology itself, we further examined only ChIP-seq and ChIP-PET data to reproduce the network (Figure 6). We found a total of 23 TFs in the network in which there is about 50% increase in terms of the number of nodes. After comparing the combined network (ChIP-seq and ChIP-PET) with ChIP-seq network (Additional file 4, Figure S3A) or ChIP-PET network (Additional file 4, Figure S3B), about 60% of the TFs were overlapped. However, this number is only 40% for the combined network of three technologies together.

The hierarchical regulatory network analysis with DREM and Cytoscape revealed that RORA could be a potential ERα partner, which is consistent to other reports [45] showing that RORA interacts with ERα and enhances ER transcriptional activity in breast cancer. This finding also indicates that RORA was required for a subset of E2-mediated up-regulated genes associated with functions of Lyase activity. Our results may provide the guidance for further investigation on the role of this co-regulation pathway played in breast cancer.

In MCF7-T cells, we found very small proportion (21.2%) of differential expressed genes overlapped with MCF7 cells (ChIP-seq dataset) with both ERα and Pol-II binding peaks. In addition, the number of common TFs of the network between MCF7-T and MCF7 cells is only 25%. Moreover, we also found two TFs with opposite expression trend between MCF7 and MCF7-T cells, in which MSX2 is up-regulated in MCF7-T cells and down-regulated in MCF7 cells, whereas ESR1 (ERα) is down-regulated in MCF7-T cells. We also identified 3 new Hub TFs in MCF7-T cells which were not found in MCF7 cells. This indicates that ERα may be no longer the most important transcription factor, other transcriptional regulators and signalling pathways may play important role in tamoxifen resistant MCF7 cells. Our comparative analysis also suggests that the ERα associated regulatory network in MCF7-T cells is rewired upon E2 induced.

We have performed additional experiments using both RT-qPCR and ChIP-qPCR to validate randomly select eight ER regulated binding loci in MCF7-T cell, and the result demonstrated that the predicted ER regulated binding loci with differential gene expression after E2 treatment were validated as shown in Additional file 10, Figure S5.

We further compared the E2 induced gene functions to specify the difference between MCF7 and MCF7-T cell line. We selected the genes that were differentially expressed and with ERα and PolII binding site. These genes were performed by GO function by the DAVID program [46] and the top 10 (the close functions were removed) categories were selected based on the *p*-value. The result was shown in Additional file 11, Figure S6 and Additional file 12, Table S6. 4 of the 10 functions were the same between those two groups of genes.

## Conclusions

In summary, we analyzed hierarchical regulatory networks for estrogen-dependent regulation in MCF7 and MCF7-T cells. We systematically compared different ChIP-based technologies as well as different breast cancer cells. Our results revealed extended hierarchal regulatory networks with new target hubs in breast cancer cells. Our computational analytical approach may also provide a framework for dissecting transcriptional regulatory networks in response to breast cancer and other human diseases.

## Methods

**Figure 7:**
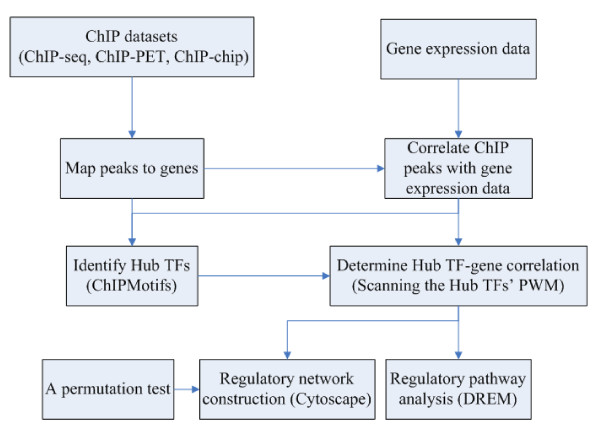
**A summary of the computational analytical approach**.

Our computational analytical approach (Figure 7) started with ChIP based datasets and gene expression data. The identified binding peaks of a given TF and Pol-II are then located to known genes, and genes having both the given TF and Pol-II binding peaks are further correlated with gene expression data based on RefSeq Gene ID. The given TF binding peaks are further used for finding the most significant motifs by the ChIPMotifs, in which they are used as Hub TFs. The Hub TF-gene connection is determined by scanning the Hub TFs' PWMs in all binding peaks and a permutation test (described in detail in **Methods **section "ERα regulatory network analysis") is used to test the reliability of each connection of the network. The resulted regulatory network is visualized by Cytoscape and the pathway is analyzed by DREM. In this study, a given TF is ERα.

### ChIP and microarray data of MCF7 cells

For MCF7 cells, the ChIP-chip data is from Carroll's paper [17], ChIP-seq data is from Welboren's paper [18], and ChIP-PET data is from Melissa's paper [19].

The time-series E2-induced gene expression data in MCF7 cells for all three ChIP-based datasets was obtained from Carroll et al. 2006 [17]. The data was with E2 treatment for 4 time points: 0 hr, 3 hr, 6 hr and 12 hr. We considered those genes were up-regulated if the gene expression values of time point 12 hr are bigger than time point 0, and verse versa.

### ChIP and microarray data of MCF7-T cells

ChIP-seq of E2-induced MCF7-T cells were maintained in a hormone-free medium (phenol red-free MEM with 2 mmol/L L-glutamine, 0.1 mmol/L nonessential amino acids, 50 units/mL penicillin, 50 Ag/mL streptomycin, 6 ng/mL insulin, and 10% charcoal-stripped FBS) supplemented with 10^-7 ^mol/L 4-hydroxytamoxifen. Prior to all experiments, MCF7-T cells were cultured in hormone-free medium for 1 week to deplete any residual OHT, and then MCF7-T cells were cultured in hormone-free basal medium (phenol-red free MEM with 2 mM L-glutamine, 0.1 mM non-essential amino acids, 50 units/ml penicillin, 50 μg/ml streptomycin, and 3% charcoal-dextran stripped FBS) for three days. MCF7-T cells were treated with E2 (108 mol/L) for 3 hours.

5 × 10^7 ^cells were crosslinked with 1% formaldehyde for 10 min, at which point 0.125 M glycine was used to stop the crosslinking. In brief, after crosslinking, cells were treated by lysis buffers and sonicated to fragment the chromatin to a size range of 200 bp-1 kb. Chromatin fragments were then immunoprecipitated with 10 ug of antibody/magnetic beads. The antibodies against Pol-II and ERα were purchased from Santa Cruz Biotechnology (Santa Cruz, sc-899 × and sc-8005 X). After immunoprecipitation, washing, and elution, ChIP DNA was purified by phenol:chloroform:isoamyl alcohol and solubilized in 70 μl of water. Then Illumina library was constructed and sequenced with Illumina/Solexa Genome Analyzer (Canada's Michael Smith Genome Sciences Centre, Vancouver, CA). Briefly, the ChIP DNA sample was run in 12% PAGE and the 100-300 bp DNA fraction was excised and eluted from the gel slice overnight at 4°C in 300 μl of elution buffer (5:1, LoTE buffer (3 mM Tris-HCl, pH 7.5, 0.2 mM EDTA)-7.5 M ammonium acetate) and was purified using a QIAquick purification kit (Qiagen, Cat#28104). The library was constructed using Illumina genomic DNA prep kit by following its protocol (Illunima, cat# FC-102-1002), clusters were generated on the Illumina cluster station (Illumina, cat# FC-103-1002), and the sequence was run on Illumina 1G Analyzer following the manufacturer's instructions (Illumina, cat# FC-104-1003). The unique mapped ChIP-seq datasets (bed format, total four files: control using ER antibody, E2 treated using ER antibody, control using Pol-II antibody and E2 treated using Pol-II antibody) for MCF7-T cells are available from GEO database (accession number: GSE26083) or from our webpage http://motif.bmi.ohio-state.edu/ERNetwork/.

For E2-induced gene expression data in MCF7-T cells, Affymetrix U133 Plus2.0 array platform consisting of ~55,000 transcripts was used for measuring E2-induced gene expression in MCF7-T cells. Three replicates from different biological samples were performed and normalized using MASS5 in the statistic package R http://www.R-project.org (Additional file 13, Table S7). A p-value less than 0.05 using a Welch's t-test and 2 fold changes for each gene were used as cutoff thresholds to determine a set of differential expressed genes. Finally, we mapped the genes with Human TFs and get 1,667 genes remains.

### RT-qPCR and ChIP-qPCR

After 3 hr of E2 treatment, control and treated MCF7-T cells were subjected to total RNA extraction by Trizol reagent (Invitrogen). Total RNA (2 μg) was reverse transcribed to cDNA with oligo-dT (SuperScript III; Invitrogen). Quantitative RT-PCR was performed by using SYBR Green dye chemistry (Applied Biosystems) on a 7500 Real-time PCR system apparatus (Applied Biosystems). Gene Expression was measured by the ΔΔCt method using *GAPDH *as the internal control. Statistical analyses were carried out by using a two-tailed t test. Specific primers for amplification are available on request.

To confirm candidate ERα target genes determined by ChIP-seq, PCR primers targeting a region within 200 bp of the predicted ERE were used to measure the enrichment of this sequence in anti-ERa-immunoprecipitated samples by quantitative PCR with SYBR Green-based detection method (Applied Biosystems). Quantitative values measured by a standard curve (50 to 0.08 ng, 5-fold dilution, R2 > 0.99) of input DNA amplified with the same primer set. Results are presented as the mean of triplicates with standard derivation.

### Calling peaks of ChIP-based data

A standard procedure for extracting image files, mapping the reads onto human genome, and filtering the mapped reads to unique reads was followed with the Solexa 1.6 pipeline [47]. Only uniquely mapped reads with a length of 36 bp were then used further for determining the binding regions by our BELT program [48]. For all datasets, we narrowed the peaks with 500 bp in length. All the common peak number in Figures 1 and 5A were overlapped according to the gene ID of each file. We used the fisher exact test [49] to calculate the *p*-value of each overlapped number, and assumed that the human genome number is 30,000 (Additional file 14, Table S8).

### ERα regulatory network analysis

The following steps were used to calculate the position weight matrix (PWM).

Suppose there are m sequences, and the sequence length is n. For each column j(1 ≤ *j *≤ *n*), the occurrence frequency of every nucleotide (A or T or C or G) was calculated by counting the number in m sequences (equation 1).

(1) $$f_{i,j}=\frac{\sum_{i=1}^{m}\left(S_{i,j}=c\right)}{m}$$

Where c is one of base type (A/T/C/G), S_i,j _is the nucleotide at row i, column j.

Hence, the PWM is represented as a 4*n matrix. For row i(1 ≤ *i *≤ 4) and column j(1 ≤ *j *≤ *n*), the PWM can be calculated:

(2) $$w(i,j)=\log_{2}\frac{f_{i,j}}{p(i)}$$

Where p(i) is the background frequency of nucleotide i. For Human genome, p(i = A) = p(T) = 30% and p(i = C) = p(G) = 20%, approximately.

For the case that the number sample is small, the equation 1 needed to be replaced as

(3) $$f_{i,j}=\frac{\sum_{i=1}^{m}\left(S_{i,j}=c\right)+\frac{\sqrt{m}}{4}}{m+\sqrt{m}}$$

Then, the significant PWMs were mapped to TransFac [36] database and JASPAR [37] database to find the most similar TFs. And ERα and its partner TFs were found.

To construct the regulatory network, the PWM was used to scan the peak region of each gene. To make the result more reliable, we use stringent threshold (1 for core score, 0.95 for PWM score) to determine the underlying TF binding site. The PWM score and core score is calculated as follows:

Given a sequence with the same length (column number n) of PWM, we can first calculate the sequence score:

(4) $$S_{seq}=\sum_{j=1}^{n}w(S_{i},j)$$

Where *w*(*S_i_*, *j*) is the score at row i which the nucleotide is the same as the given sequence at column j.

Then, the minimum and maximum score of PWM can be calculated as:

(5) $$S_{\min}=\sum_{j=1}^{n}\min\{w(i,j)\}$$

(6) $$S_{\min}=\sum_{j=1}^{n}\min\{w(i,j)\}$$

The PWM score for the given sequence is shown in equation 7

(7) $$S_{PWM}=\frac{S_{seq}-S_{\min}}{S_{\max}-S_{\min}}$$

Our program ChIPMotifs will give the length (k) and start (k1) and end (k2) position of Core region in a PWM. The given sequence will be scanned (n-k+1) times (move forward one position each time) to find a maximum Core score. For the dth time, the sequence score can be represented as:

(8) $$S_{seq,d}=\sum_{j=d}^{d+k-1}w(S_{i},j)$$

For equation 5 and 6, only the boundary of j was changed from 1-n to k1- k2. The Core score for the dth time is

(9) $$S_{Core,d}=\frac{S_{seq,d}-S_{\min}}{S_{\max}-S_{\min}}$$

And the Core score for the given sequence is

(10) $$S_{Core}=\underset{d}{\max}(S_{Core,d})$$

If there is Motif for ERα and Hub TFs for certain gene, a connection between the Hub TF (include ERα) and the gene was made. We further facilitated these relationships by visualizing an estrogen regulatory network with Cytoscape [42] software platform (see Results section).

To test the significance of the network proposed in this study, a statistical strategy (permutation) was used to determine the probability of each edge of the network under random circumstances. Since the TF binding site region is composed of specific sequences, and only by scanning the sequence region using PWM, we get the network edges. Hence, we shuffled the sequence of each peak region for 1000 times, and to see how many times a specific TF binding site is hit by the scanning process. The ratio (times hit by scanning divide by 1000) of each edge was calculated. The number with low value was considered high statistical significance (we used 0.2 as a cutoff to include as more connection as possible while keeping the relationship reliable).

### ERα regulatory pathway analysis

The up/down-regulated genes were grouped into several classes (sub up/down-regulated classes). For each group, the common gene functions were identified from GO database with p-value represented for the significance. Then, we used the PWMs of the 6 TFs (ERα, RORA, FOS, FOXA1 PITX2 and CEBP) to identify all possible binding sites in all genes with peaks. Thus we established the relationship between Hub TFs and grouped genes. A DREM diverging score less than 0.1 was used as a significant score threshold for TF-grouped gene relationship.

## Authors' contributions

FG, HKH, PYH, JW, YM, JP, THMH and VXJ conceived and designed the study. VXJ and FG performed the computational analysis and HKH, PYH and JW performed MCF7-T experiments and RT-, ChIP-qPCR validations. VXJ, FG, JP and THMH drafted the manuscript. All authors provided comments on the paper and read and approved the final manuscript.

## Supplementary Material

Additional file 1 **Table S1**. A distribution of locations of Pol-II and ERα binding sites relative to Human HG18 RefSeq Genes three ChIP-based datasets in MCF7 cell line. Click here for file

Additional file 2 **Figure S1. A plot of the distribution of identified ERα and Pol-II binding loci relative to a known gene's TSS**. A) Definition of different regions of a gene. B) The histogram of the distribution of peak location. A big portion of Pol-II bind in promoter regions (2 kb around TSS); A small portion of ERα binding loci are located in promoter region. These observations confirm that the majority of ERα binding loci are outside of proximal promoter regions in which it is consistent with the results from other studies. Click here for file

Additional file 3 **Figure S2. Three major binding models for ERα regulated gene expression**. A) direct binding to ERE (estrogen response element); B) indirect binding, through which it binds to other TF partners which bind to DNA; C) co-occurrent binding, where both ERα and other TF partners bind to their own specific DNA motifs. Click here for file

Additional file 4 **Figure S3. The Regulatory network for E2 treated MCF7 cell line of three different ChIP-based datasets**. A) The Regulatory network for E2 treated MCF7 cell line from ChIP-seq dataset. B) The Regulatory network for E2 treated MCF7 cell line from ChIP-PET dataset. C) The Regulatory network for E2 treated MCF7 cell line from ChIP-chip dataset. Click here for file

Additional file 5 **Table S2**. The list of common TFs among all three regulatory networks. Click here for file

Additional file 6 **Figure S4. Regulatory pathway analysis from three ChIP-based dataset. Figure S4 (I). Regulatory pathway analysis from ChIP-seq dataset**. A) The time series gene expression data of E2 induced genes superimposed with the regulatory pathway map produced by DREM using the gene expression profile as well as ERα binding sites and PolII binding sites. The bright green nodes indicate split points where the sets of expression of genes diverge. B) Paths out of splits are annotated with TFs determined by DREM to be associated with the genes assigned to the path at a score <0.1. The GO annotations for the genes in 5 of the paths are shown at the right with their p-values. C) The genes traversing the 3 splits are shown with (a) corresponding to the split at 0-hr, (b) corresponding to the split at 3-hr and (c) corresponding to the split at 6-hr. Figure S4 (II). Regulatory pathway analysis from ChIP-PET dataset. A) The time series gene expression data of E2 induced genes superimposed with the regulatory pathway map produced by DREM using the gene expression profile as well as ERα binding sites and PolII binding sites. The bright green nodes indicate split points where the sets of expression of genes diverge. B) Paths out of splits are annotated with TFs determined by DREM to be associated with the genes assigned to the path at a score <0.1. The GO annotations for the genes in 5 of the paths are shown at the right with their p-values. C) The genes traversing the 3 splits are shown with (a) corresponding to the split at 0-hr, (b) corresponding to the split at 3-hr and (c) corresponding to the split at 6-hr. Figure S4 (III). Regulatory pathway analysis from ChIP-chip dataset. A) The time series gene expression data of E2 induced genes superimposed with the regulatory pathway map produced by DREM using the gene expression profile as well as ERα binding sites and PolII binding sites. The bright green nodes indicate split points where the sets of expression of genes diverge. B) Paths out of splits are annotated with TFs determined by DREM to be associated with the genes assigned to the path at a score <0.1. The GO annotations for the genes in 5 of the paths are shown at the right with their p-values. C) The genes traversing the 3 splits are shown with (a) corresponding to the split at 0-hr, (b) corresponding to the split at 3-hr and (c) corresponding to the split at 6-hr. Click here for file

Additional file 7 **Table S3**. A. The five paths for ERα regulated genes identified by DREM analysis in ChIP-seq data. B. The five paths for ERα regulated genes identified by DREM analysis in ChIP-PET data. C. The five paths for ERα regulated genes identified by DREM analysis in ChIP-chip data. Click here for file

Additional file 8 **Table S4**. A. The transcription factors identified in ChIP-seq with predicted binding motifs. B. The transcription factors identified in ChIP-PET with predicted binding motifs. C. The transcription factors identified in MCF7 with predicted binding motifs. Click here for file

Additional file 9 **Table S5**. Transcription factor network identified by ReMoDiscovery. Click here for file

Additional file 10 **Figure S5. Validation of the regulatory network of MCF7-T cell line**. 8 TFs were selected from MCF7-T network. All the TFs were found with ERα binding peaks. (A) mRNA levels derived from RT-qPCR of 8 ERα regulated target genes were shown under E2 (10 nM, 3hr) stimulation in MCF7-T cells. GAPDH was as internal control. Mean ± SD (n = 3). (B) Validations of predicted ERα-binding regions for 8 binding loci by ChIP-qPCR. Control and E2-treated MCF7-T cells were subjected to ChIP-qPCR with ERα antibody. Mean ± SD (n = 3). Click here for file

Additional file 11 **Figure S6. A comparison of GO functions between MCF7 and MCF7-T cells using genes from ChIP-seq technology**. (A) GO functions of MCF7 cells. (B) GO functions of MCF7-T cells. (C) the GO function of two cells in the same figure. Click here for file

Additional file 12 **Table S6**. A list of genes used for GO function analysis for MCF7 and MCF7-T ChIP-seq data. Click here for file

Additional file 13 **Table S7**. Gene expression data of MCF7-T cells. Click here for file

Additional file 14 **Table S8**. Fisher exact test of the significance of overlapped genes in Figures 1A and 5A. Click here for file
