# Supplementary material for: Inference of hierarchical regulatory network of estrogen-dependent breast cancer through ChIP-based data
Source: BMC Syst Biol. 2010 Dec 17;4:170. doi: 10.1186/1752-0509-4-170 (PMC3012048; doi:10.1186/1752-0509-4-170)
Supplement: Additional file 2 — Figure S1. A plot of the distribution of identified ERα and Pol-II binding loci relative to a known gene's TSS. A) Definition of different regions of a gene. B) The histogram of the distribution of peak location. A big portion of Pol-II bind in promoter regions (2 kb around TSS); A small portion of ERα binding loci are located in promoter region. These observations confirm that the majority of ERα binding loci are outside of proximal promoter regions in which it is consistent with the results from other studies. [file 1752-0509-4-170-S2.PPT]

## Slide 1
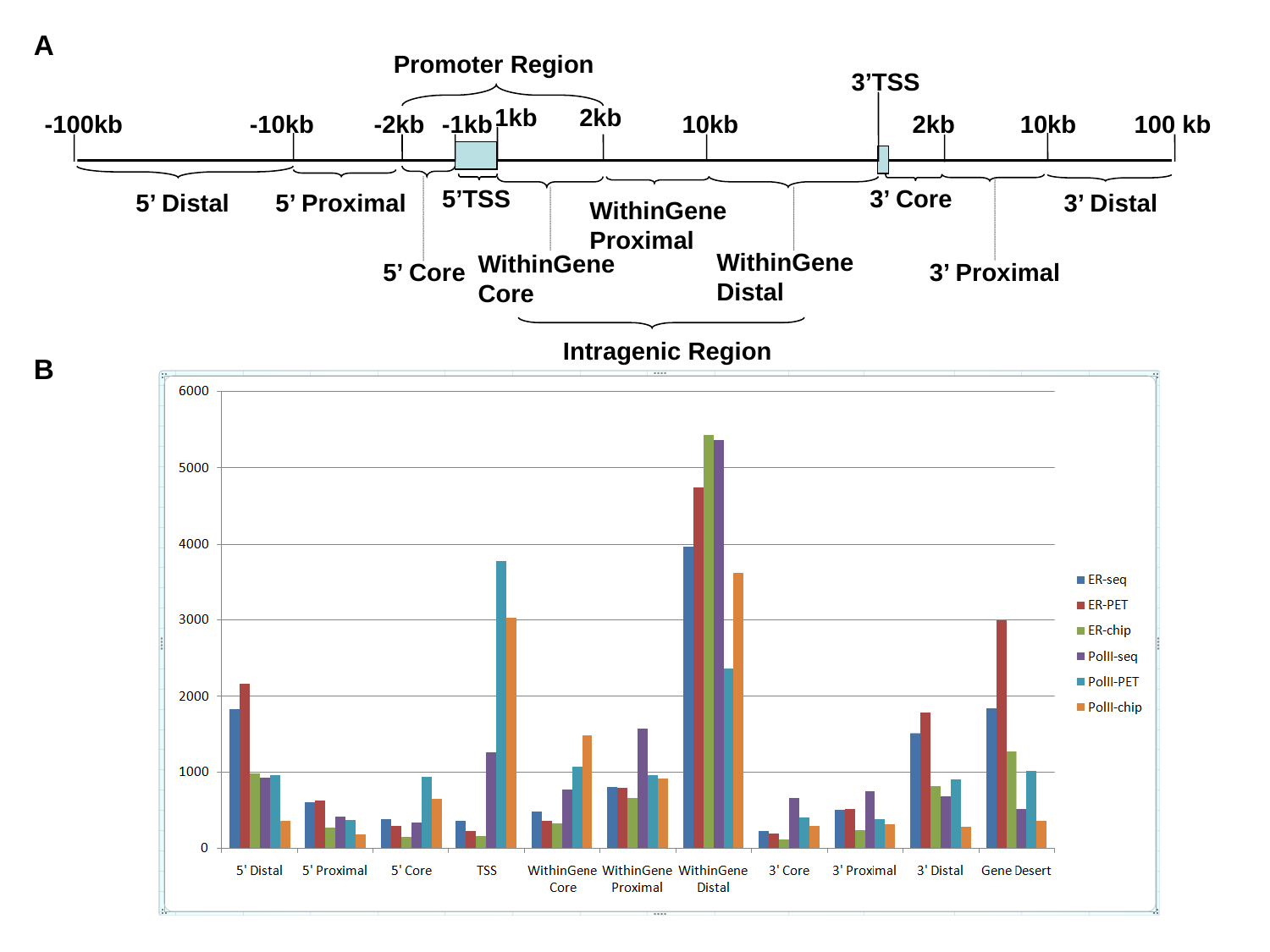

A
Promoter Region
3’TSS
-100kb
-10kb
10kb
100 kb
5’TSS
5’ Distal
5’ Proximal
3’ Distal
WithinGene
Core
3’ Proximal
1kb
2kb
-2kb
-1kb
10kb
2kb
3’ Core
WithinGene
Proximal
WithinGene
Distal
5’ Core
Intragenic Region
B
