# Supplementary figures and images for: Inference of hierarchical regulatory network of estrogen-dependent breast cancer through ChIP-based data
Source: BMC Syst Biol. 2010 Dec 17;4:170. doi: 10.1186/1752-0509-4-170 (PMC3012048; doi:10.1186/1752-0509-4-170)

## Slide 1
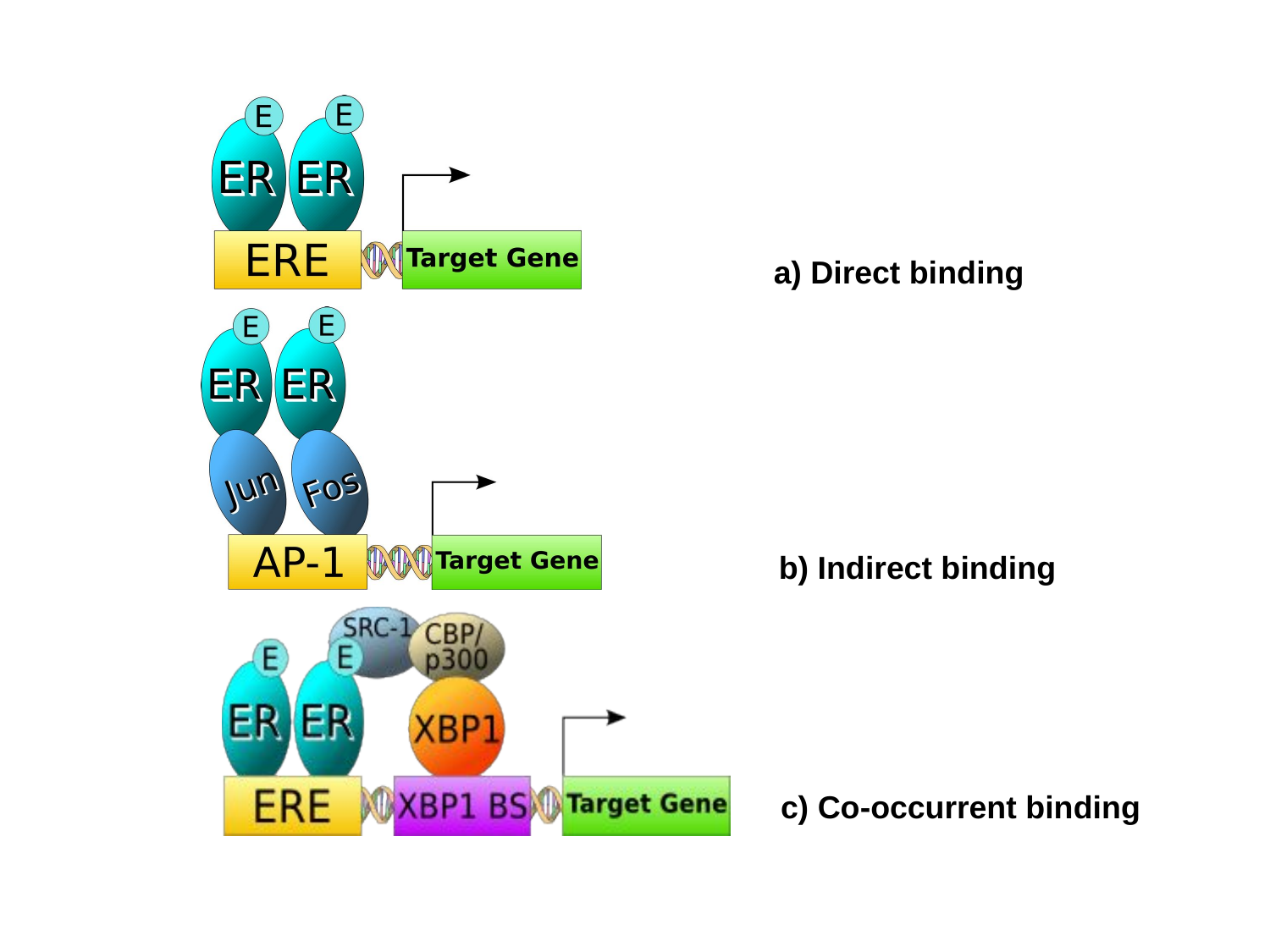

a) Direct binding
b) Indirect binding
c) Co-occurrent binding

Supplement: Additional file 3 — Figure S2. Three major binding models for ERα regulated gene expression. A) direct binding to ERE (estrogen response element); B) indirect binding, through which it binds to other TF partners which bind to DNA; C) co-occurrent binding, where both ERα and other TF partners bind to their own specific DNA motifs. [file 1752-0509-4-170-S3.PPT]

## Slide 1
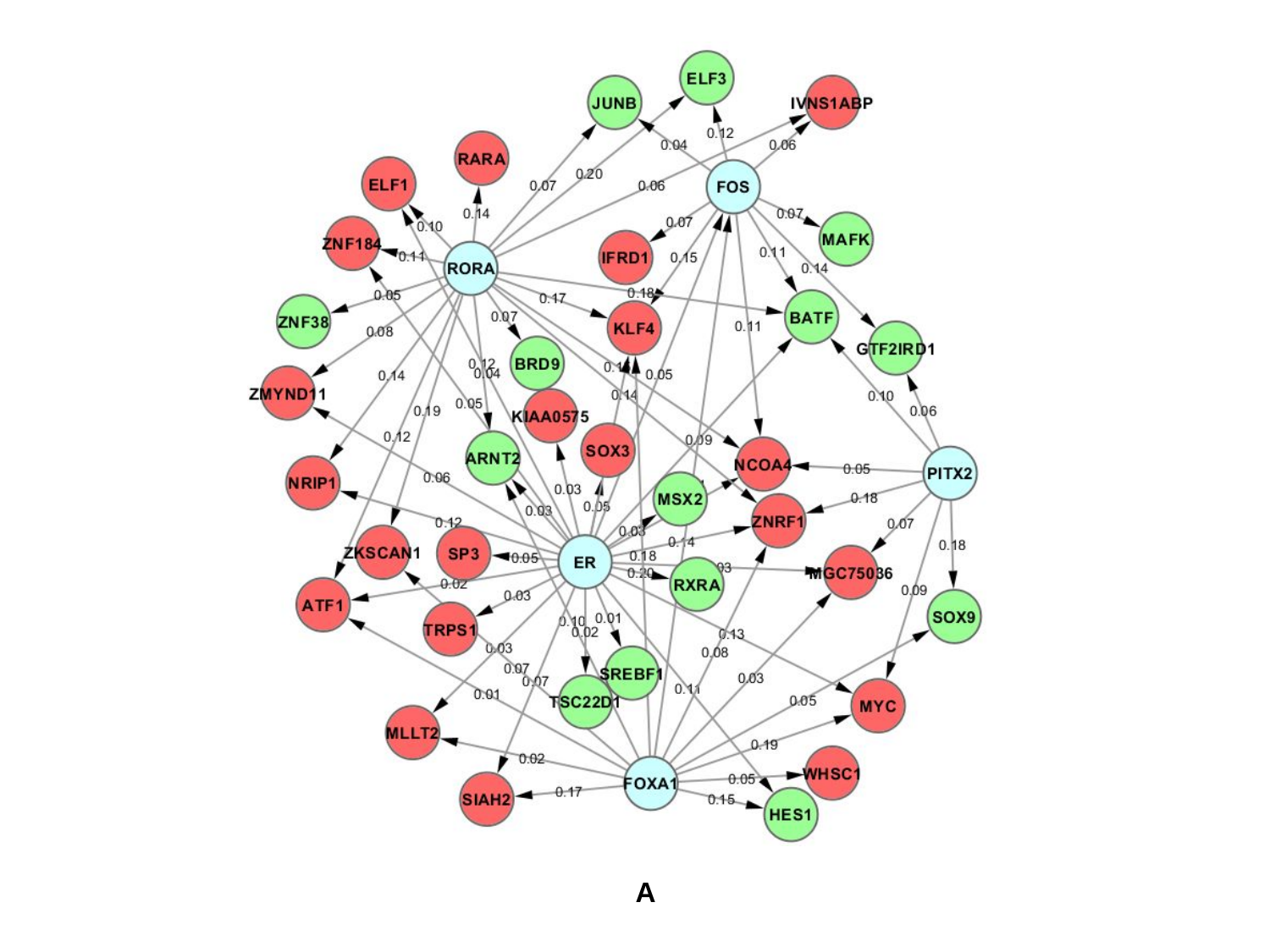

A

## Slide 2
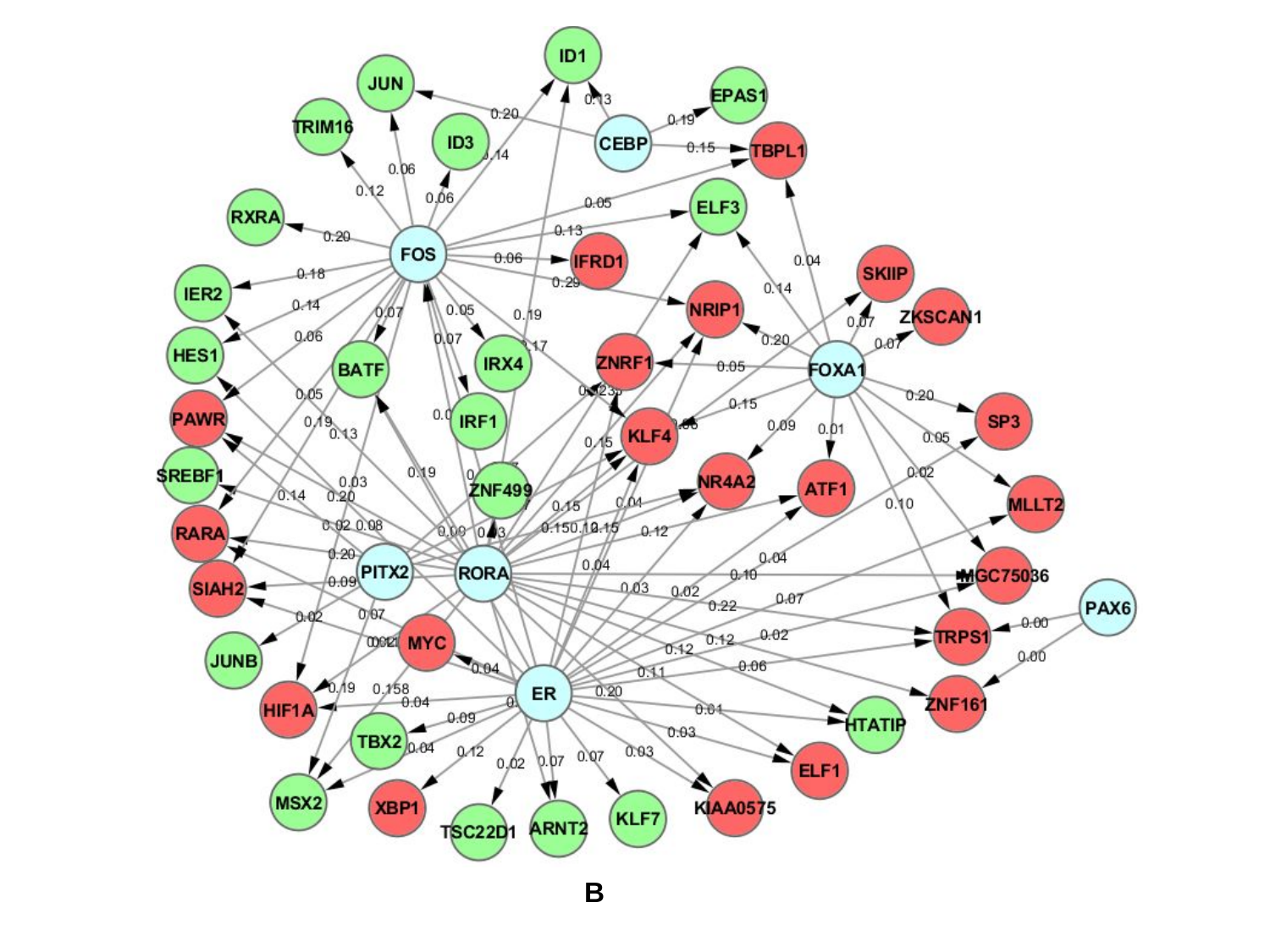

B

## Slide 3
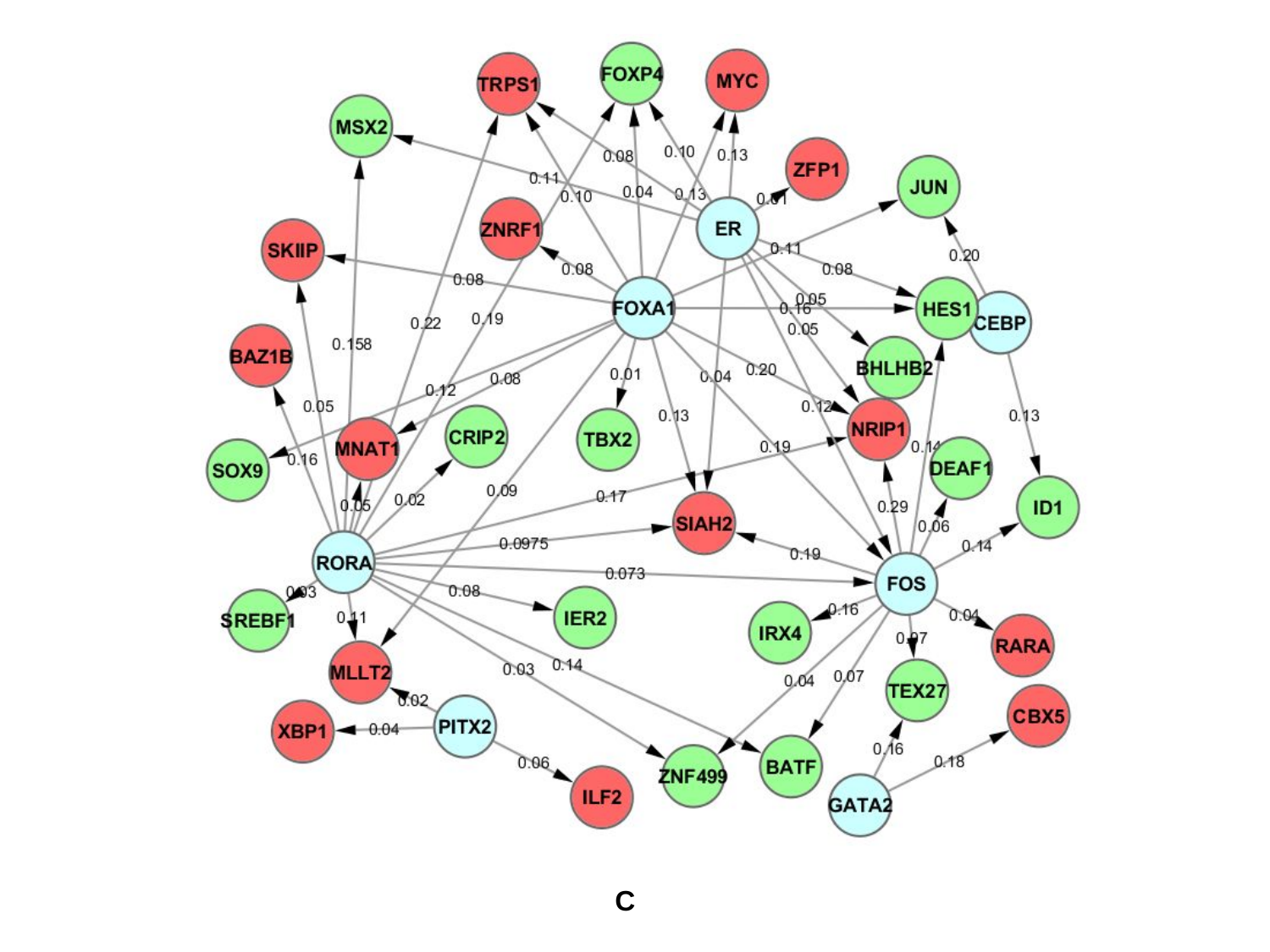

C

Supplement: Additional file 4 — Figure S3. The Regulatory network for E2 treated MCF7 cell line of three different ChIP-based datasets. A) The Regulatory network for E2 treated MCF7 cell line from ChIP-seq dataset. B) The Regulatory network for E2 treated MCF7 cell line from ChIP-PET dataset. C) The Regulatory network for E2 treated MCF7 cell line from ChIP-chip dataset. [file 1752-0509-4-170-S4.PPT]

## Slide 1
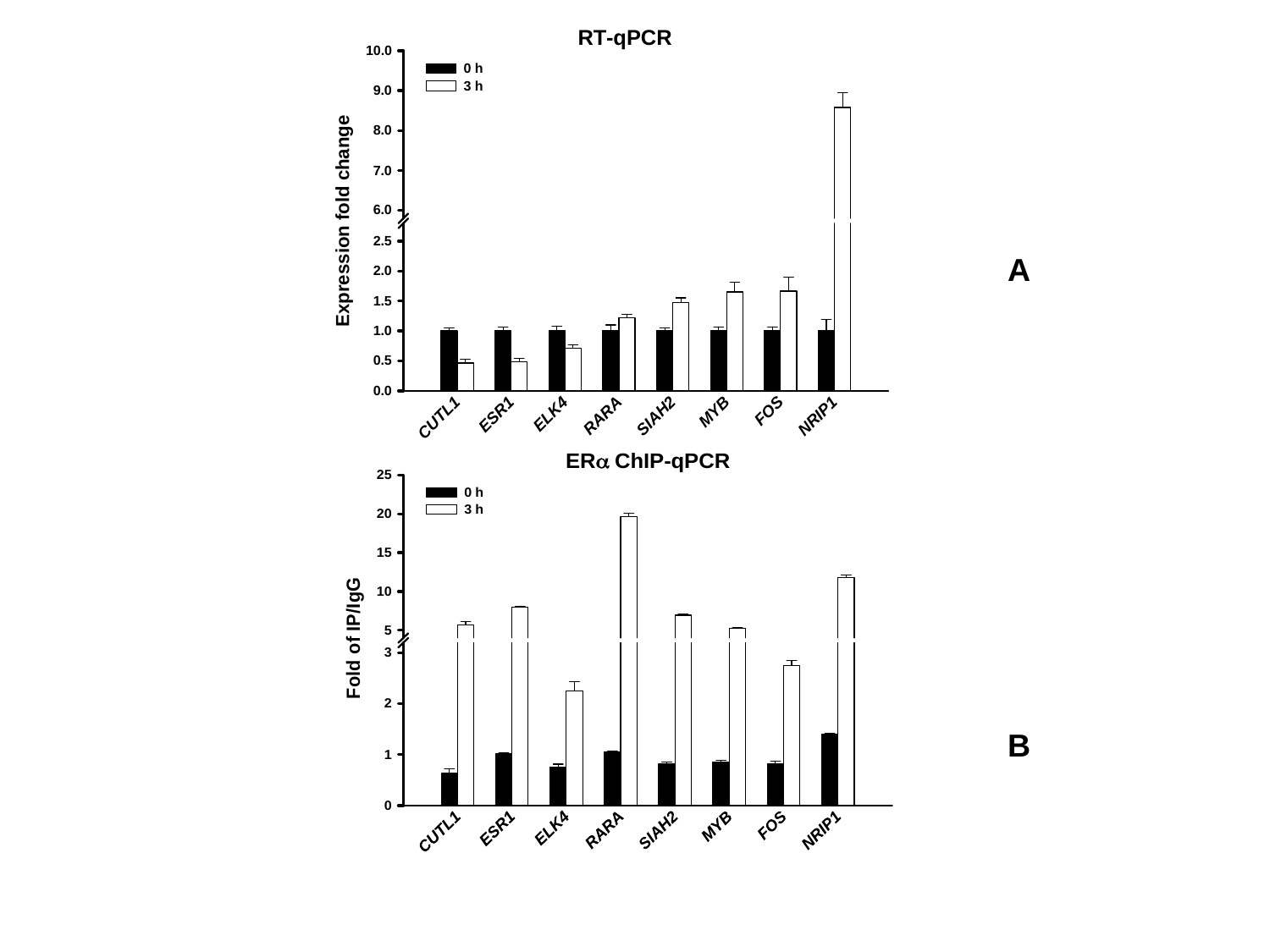

A
B

Supplement: Additional file 10 — Figure S5. Validation of the regulatory network of MCF7-T cell line. 8 TFs were selected from MCF7-T network. All the TFs were found with ERα binding peaks. (A) mRNA levels derived from RT-qPCR of 8 ERα regulated target genes were shown under E2 (10 nM, 3hr) stimulation in MCF7-T cells. GAPDH was as internal control. Mean ± SD (n = 3). (B) Validations of predicted ERα-binding regions for 8 binding loci by ChIP-qPCR. Control and E2-treated MCF7-T cells were subjected to ChIP-qPCR with ERα antibody. Mean ± SD (n = 3). [file 1752-0509-4-170-S10.PPT]
