# Supplementary material for: Inference of hierarchical regulatory network of estrogen-dependent breast cancer through ChIP-based data
Source: BMC Syst Biol. 2010 Dec 17;4:170. doi: 10.1186/1752-0509-4-170 (PMC3012048; doi:10.1186/1752-0509-4-170)
Supplement: Additional file 6 — Figure S4. Regulatory pathway analysis from three ChIP-based dataset. Figure S4 (I). Regulatory pathway analysis from ChIP-seq dataset. A) The time series gene expression data of E2 induced genes superimposed with the regulatory pathway map produced by DREM using the gene expression profile as well as ERα binding sites and PolII binding sites. The bright green nodes indicate split points where the sets of expression of genes diverge. B) Paths out of splits are annotated with TFs determined by DREM to be associated with the genes assigned to the path at a score <0.1. The GO annotations for the genes in 5 of the paths are shown at the right with their p-values. C) The genes traversing the 3 splits are shown with (a) corresponding to the split at 0-hr, (b) corresponding to the split at 3-hr and (c) corresponding to the split at 6-hr. Figure S4 (II). Regulatory pathway analysis from ChIP-PET dataset. A) The time series gene expression data of E2 induced genes superimposed with the regulatory pathway map produced by DREM using the gene expression profile as well as ERα binding sites and PolII binding sites. The bright green nodes indicate split points where the sets of expression of genes diverge. B) Paths out of splits are annotated with TFs determined by DREM to be associated with the genes assigned to the path at a score <0.1. The GO annotations for the genes in 5 of the paths are shown at the right with their p-values. C) The genes traversing the 3 splits are shown with (a) corresponding to the split at 0-hr, (b) corresponding to the split at 3-hr and (c) corresponding to the split at 6-hr. Figure S4 (III). Regulatory pathway analysis from ChIP-chip dataset. A) The time series gene expression data of E2 induced genes superimposed with the regulatory pathway map produced by DREM using the gene expression profile as well as ERα binding sites and PolII binding sites. The bright green nodes indicate split points where the sets of expression of genes diverge. B) Paths out [file 1752-0509-4-170-S6.PPT]

## Slide 1
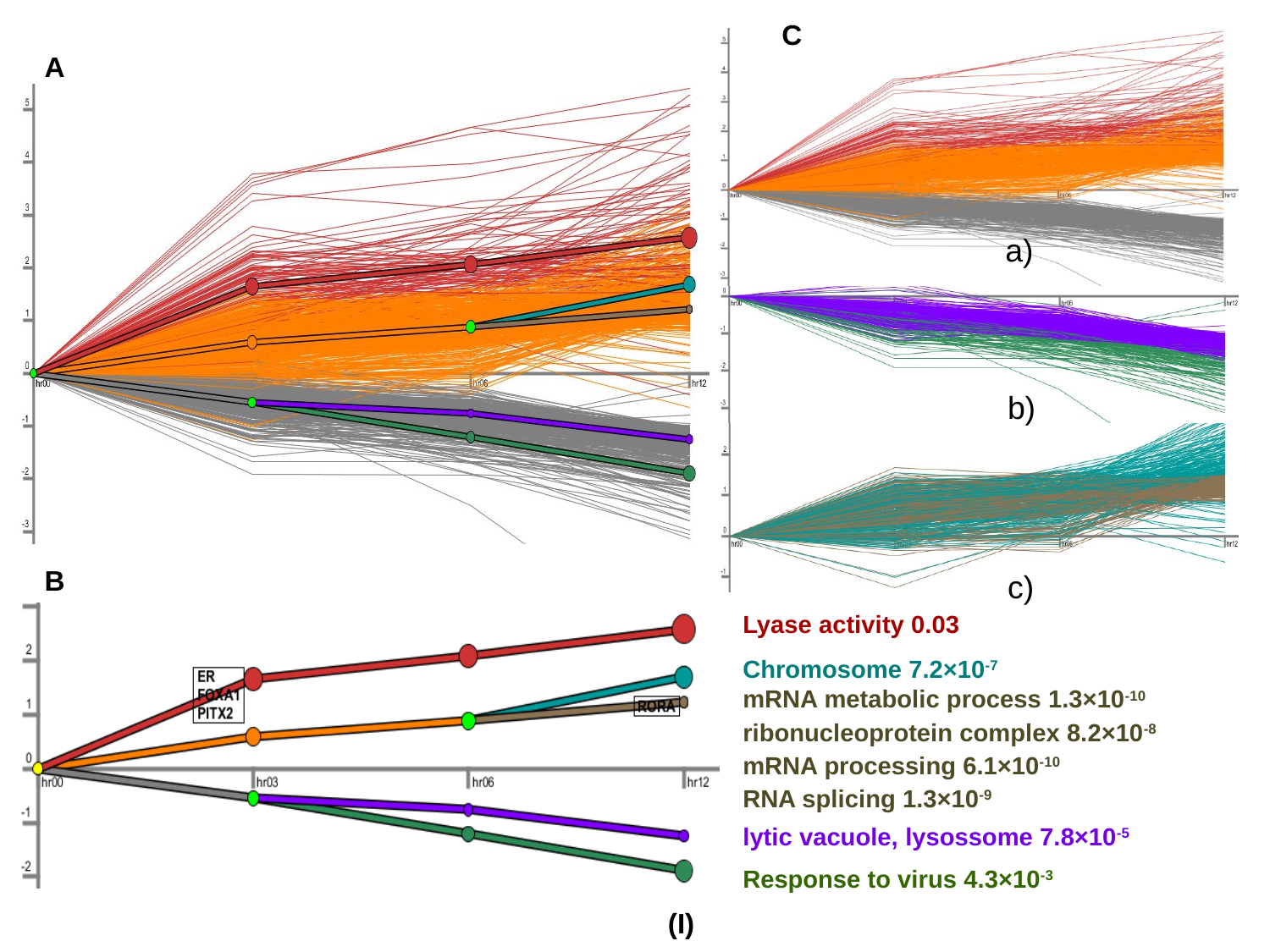

C
A
a)
b)
B
c)
Lyase activity 0.03
Chromosome 7.2×10^-7^
mRNA metabolic process 1.3×10^-10^
ribonucleoprotein complex 8.2×10-8
mRNA processing 6.1×10^-10^
RNA splicing 1.3×10^-9^
lytic vacuole, lysossome 7.8×10^-5^
Response to virus 4.3×10^-3^
(I)

## Slide 2
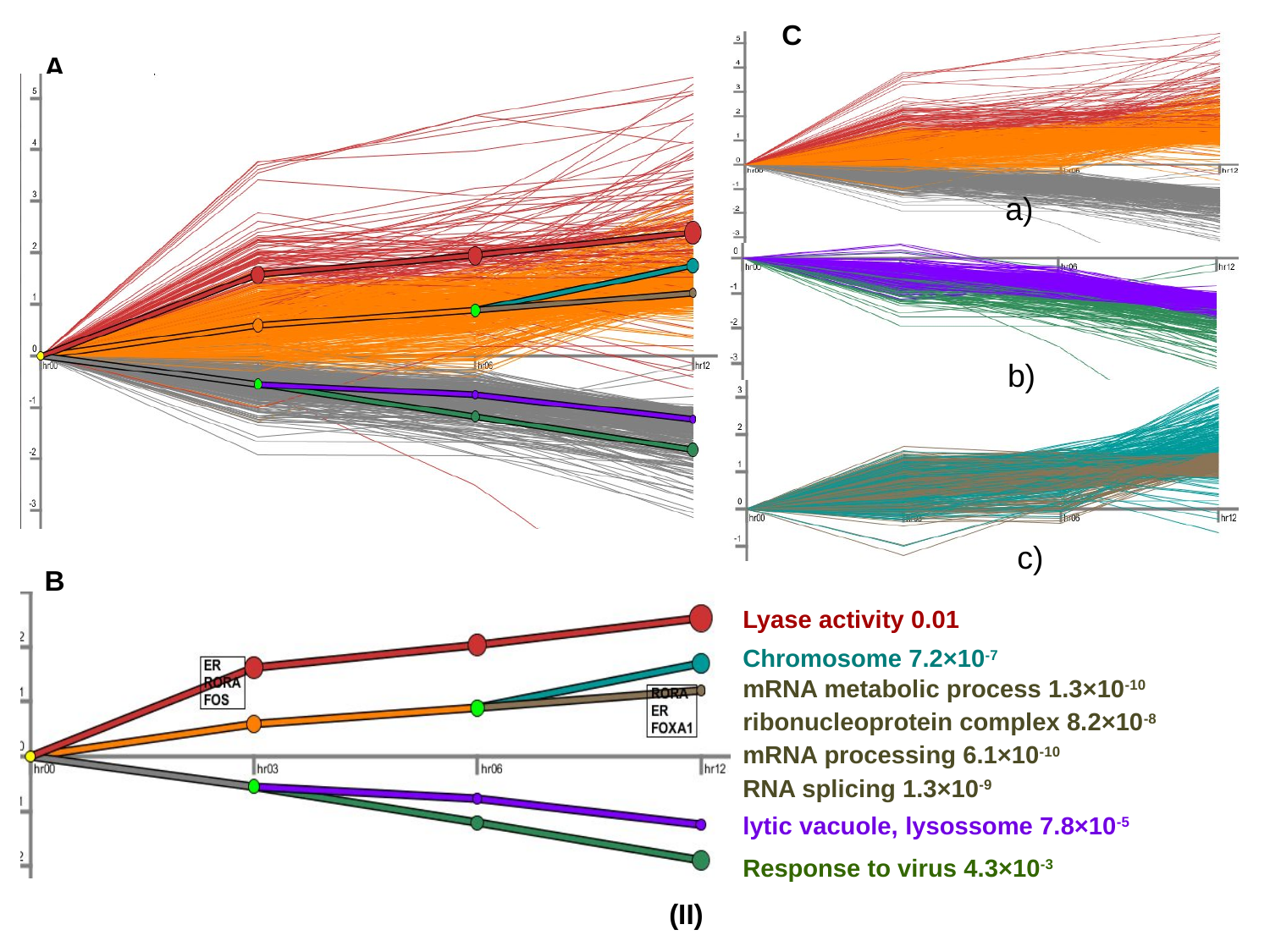

C
A
a)
b)
c)
B
Lyase activity 0.01
Chromosome 7.2×10^-7^
mRNA metabolic process 1.3×10^-10^
ribonucleoprotein complex 8.2×10-8
mRNA processing 6.1×10^-10^
RNA splicing 1.3×10^-9^
lytic vacuole, lysossome 7.8×10^-5^
Response to virus 4.3×10^-3^
(II)

## Slide 3
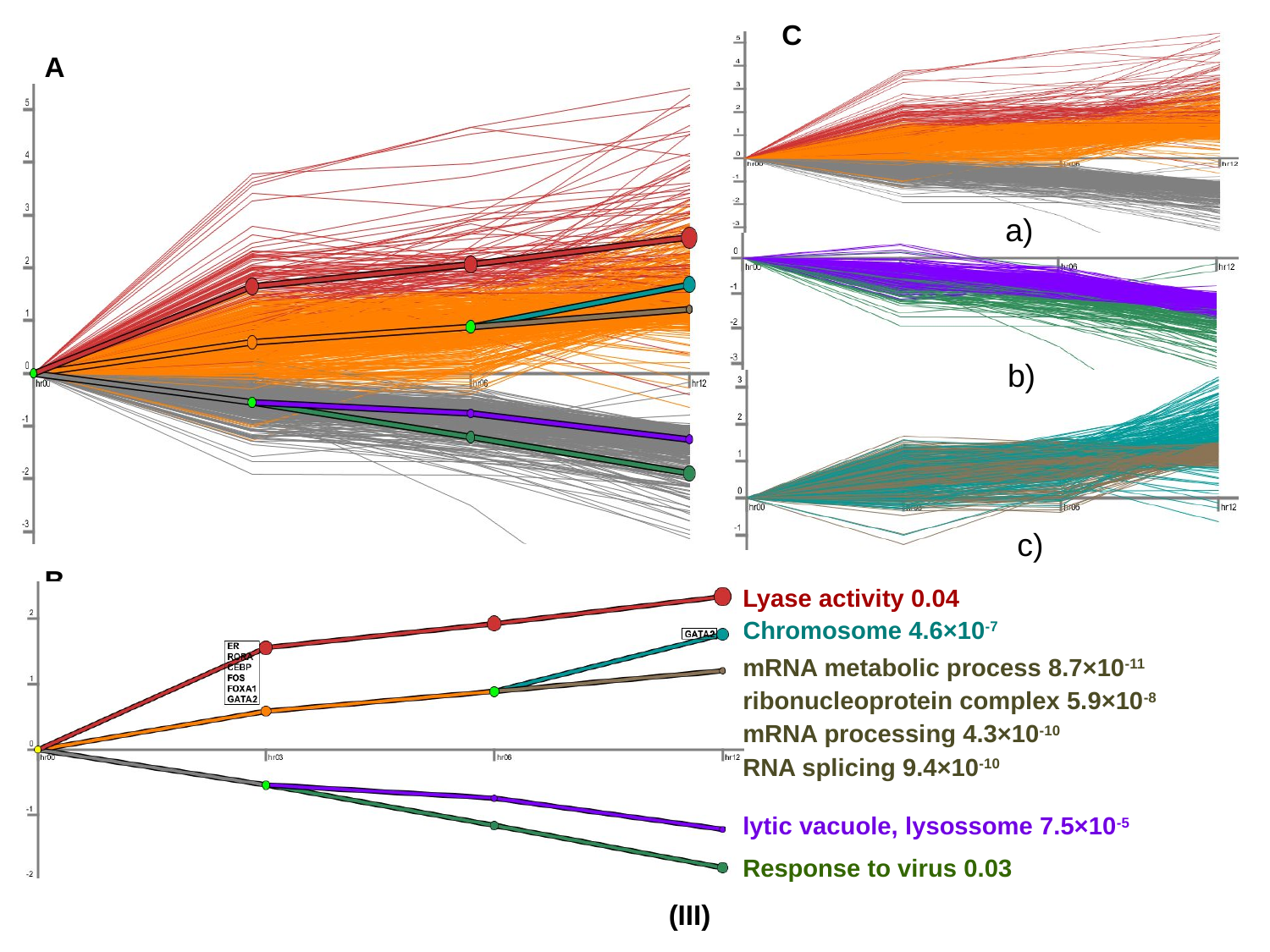

C
A
a)
b)
c)
B
Lyase activity 0.04
Chromosome 4.6×10^-7^
mRNA metabolic process 8.7×10^-11^
ribonucleoprotein complex 5.9×10-8
mRNA processing 4.3×10^-10^
RNA splicing 9.4×10^-10^
lytic vacuole, lysossome 7.5×10^-5^
Response to virus 0.03
(III)
