# Supplementary material for: Inference of hierarchical regulatory network of estrogen-dependent breast cancer through ChIP-based data
Source: BMC Syst Biol. 2010 Dec 17;4:170. doi: 10.1186/1752-0509-4-170 (PMC3012048; doi:10.1186/1752-0509-4-170)
Supplement: Additional file 11 — Figure S6. A comparison of GO functions between MCF7 and MCF7-T cells using genes from ChIP-seq technology. (A) GO functions of MCF7 cells. (B) GO functions of MCF7-T cells. (C) the GO function of two cells in the same figure. [file 1752-0509-4-170-S11.PPT]

## Slide 1
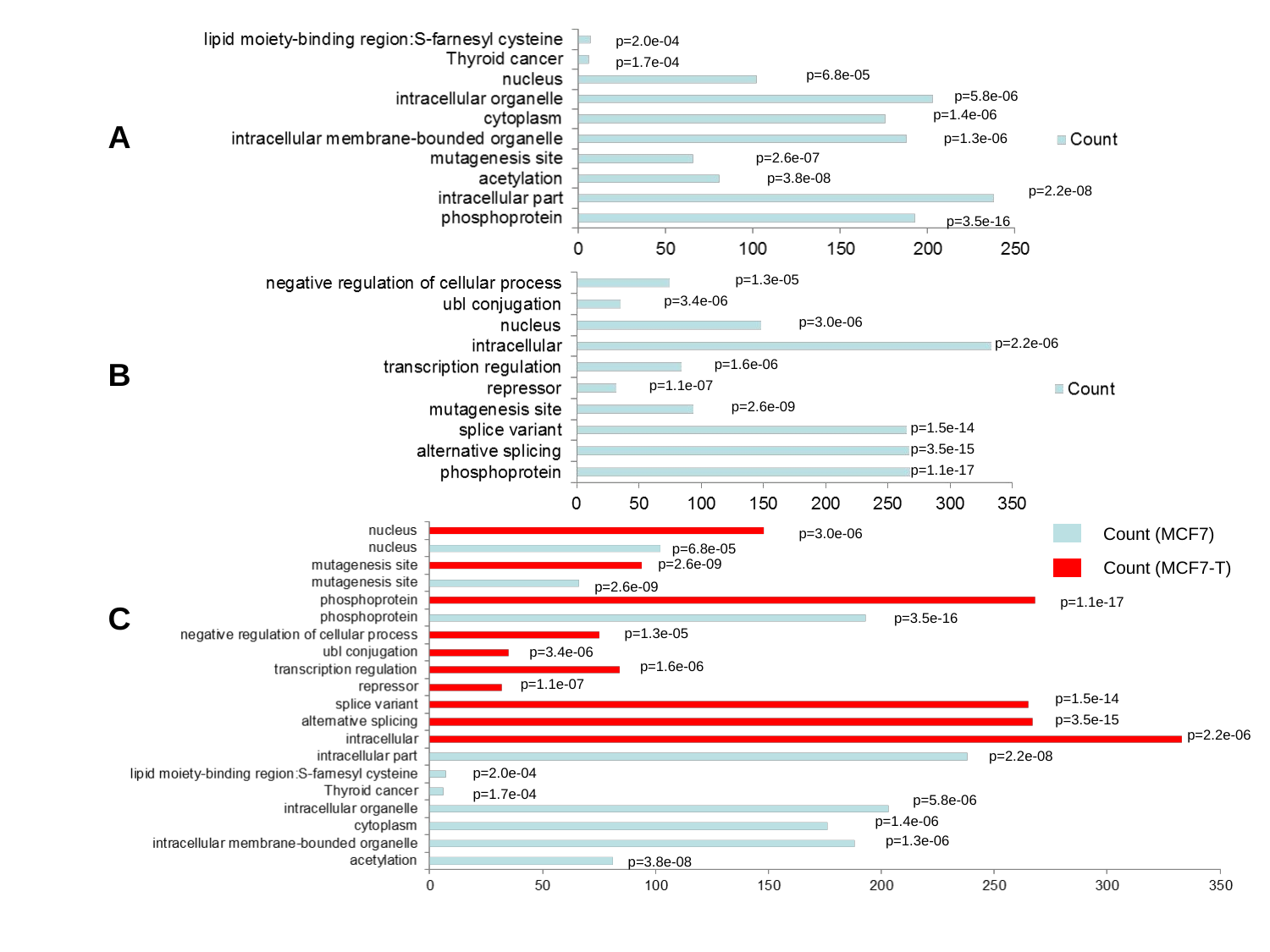

p=2.0e-04
p=1.7e-04
p=6.8e-05
p=5.8e-06
p=1.4e-06
A
p=1.3e-06
p=2.6e-07
p=3.8e-08
p=2.2e-08
p=3.5e-16
p=1.3e-05
p=3.4e-06
p=3.0e-06
p=2.2e-06
B
p=1.6e-06
p=1.1e-07
p=2.6e-09
p=1.5e-14
p=3.5e-15
p=1.1e-17
Count (MCF7)
p=3.0e-06
p=6.8e-05
p=2.6e-09
Count (MCF7-T)
p=2.6e-09
p=1.1e-17
C
p=3.5e-16
p=1.3e-05
p=3.4e-06
p=1.6e-06
p=1.1e-07
p=1.5e-14
p=3.5e-15
p=2.2e-06
p=2.2e-08
p=2.0e-04
p=1.7e-04
p=5.8e-06
p=1.4e-06
p=1.3e-06
p=3.8e-08
